# X-ray reflectivity from curved surfaces as illustrated by a graphene layer on molten copper

**DOI:** 10.1107/S1600577522002053

**Published:** 2022-04-01

**Authors:** Oleg V. Konovalov, Valentina Belova, Francesco La Porta, Mehdi Saedi, Irene M. N. Groot, Gilles Renaud, Irina Snigireva, Anatoly Snigirev, Maria Voevodina, Chen Shen, Andrea Sartori, Bridget M. Murphy, Maciej Jankowski

**Affiliations:** a ESRF – The European Synchrotron, 71 Avenue des Martyrs, 38043 Grenoble, France; b Leiden Institute of Chemistry, Leiden University, PO Box 9502, 2300 RA Leiden, The Netherlands; c Univ. Grenoble Alpes, CEA, IRIG/MEM/NRS, 38000 Grenoble, France; d Immanuel Kant Baltic Federal University, 14 Nevskogo, 236041 Kaliningrad, Russian Federation; e Deutsches Elektronen-Synchrotron DESY, Notkestrasse 85, 22607 Hamburg, Germany; f Institute for Experimental and Applied Physics, Kiel University, Olshausenstrasse 40, 24098 Kiel, Germany; g Ruprecht-Haensel Laboratory, Kiel University, Christian-Albrechts-Platz 4, 24118 Kiel, Germany

**Keywords:** X-ray reflectivity, curved surfaces, methods, synchrotron

## Abstract

The X-ray reflectivity technique can provide out-of-plane electron-density profiles of surfaces, interfaces, and thin films, with atomic resolution accuracy. While current methodologies require high surface flatness, this becomes challenging for naturally curved surfaces, particularly for liquid metals, due to the very high surface tension. Here, the development of X-ray reflectivity measurements with beam sizes of a few tens of micrometres on highly curved liquid surfaces using a synchrotron diffractometer equipped with a double crystal beam deflector is presented. The proposed and developed method, which uses a standard reflectivity θ–2θ scan, is successfully applied to study *in situ* the bare surface of molten copper and molten copper covered by a graphene layer grown *in situ* by chemical vapor deposition. It was found that the roughness of the bare liquid surface of copper at 1400 K is 1.25 ± 0.10 Å, while the graphene layer is separated from the liquid surface by a distance of 1.55 ± 0.08 Å and has a roughness of 1.26 ± 0.09 Å.

## Abstract 2

A method of X-ray reflectivity data reconstruction from scattering measurements on spherically curved surfaces is proposed and tested on solid and liquid surfaces.

## 1. Introduction

The X-ray reflectivity (XRR) technique has been successfully applied to study surfaces, interfaces, and thin films, from sub-nm to sub-µm, since the pioneering work of Heinz Kiessig (Kiessig, 1930 ▸, 1931 ▸), who identified the thickness and the decrements (δ and β) of the refractive index *n* (*n* = 1 − δ − *i*β) of a nickel layer on a glass substrate. The analytical tool developed by Lyman Parratt (Parratt, 1954 ▸) allowed further progress and to study the electron-density profiles of complex interfaces like the oxidized surface of copper. Progress in the development of X-ray sources, from the hot cathode tube to synchrotrons, provided a significant increase in beam flux, allowing for a high spatial resolution of interfacial structures on the one hand and small beams (down to few micrometers) to study smaller surfaces on the other hand. The microradian angular divergence and the high spatial coherence of the synchrotron beams allow one to study the structure of thick films (Lyatun *et al.*, 2019 ▸). Nowadays, the XRR technique can provide out-of-plane electron-density profiles with atomic resolution (Daillant & Gibaud, 1999 ▸; Tolan, 1999 ▸; Pershan & Schlossman, 2012 ▸). High surface flatness, with a curvature radius bigger than several hundred metres, is the main prerequisite for detailed studies with XRR. Although solid surfaces can be made laterally flat on a centimetre-sized sample, this task becomes challenging for liquids (deGennes *et al.*, 2002 ▸), particularly for liquid metals due to the very high surface tension (Magnussen *et al.*, 1995 ▸; Pershan & Schlossman, 2012 ▸).

One common way to reduce the liquid surface curvature problem is to use a large enough puddle, such that gravity can flatten the puddle at its center. For the case of water on an ideally hydro­phobic surface, the de Gennes formula (de Gennes *et al.*, 2002 ▸) shows that the surface is curved over several centimetres around the puddle rim. Thus, the water puddle should be a few tens of centimetres wide for a successful XRR experiment.

However, this solution is not applicable, if working with a small volume of liquid (*e.g.* for expensive materials or in a complex sample environment) would be a necessity (Shpyrko *et al.*, 2004 ▸). One of the examples of such measurement is our recent work on the growth of graphene on liquid metal catalysts (LMCats), *i.e.* molten copper, using chemical vapor deposition (CVD) (Jankowski *et al.*, 2021 ▸). The graphene is grown on the molten copper surface using a dedicated reactor specially constructed for this purpose (Saedi *et al.*, 2020 ▸). However, the intense heat of the sample at ∼1400 K, the high evaporation rate of copper, and the reactive gas environment impose some technological constraints related to the maximum sample size. For our case, the curvature radius at the center of a ∼1–2 cm-diameter puddle of liquid copper on a polished tungsten substrate would be about 100–200 mm, which is far lower than the acceptable value for current XRR methodologies.

Another solution to reduce the puddle surface curvature is to decrease the contact angle of the liquid, or in other words, to use a substrate with better liquid wettability. As will be discussed, we have succeeded in decreasing the contact angle of liquid copper on a tungsten substrate by roughening the substrate surface according to Wenzel’s equation (Wenzel, 1936 ▸). However, there is a limitation to this method, as excessive roughening would lead to substrate roughness peaks piercing out of the liquid surface, which would interfere with the XRR experiment.

An alternative way to reduce the curvature problem is to use a smaller beam (*e.g.* nano-beam instead of micro-beam mode), as this reduces the variation of the incident angle on the surface under the beam footprint (Festersen *et al.*, 2018 ▸). Recording the scattering intensity using a vertical scan of the puddle through a knife-like X-ray beam and knowing the local surface curvature at the beam position allows for reconstructing the reflectivity curve over an extensive range of scattering vector *q*_*z*_. A possible drawback of this method is that each new point of measurement is taken from a new part of the sample, which requires a good uniformity of the sample on the surface.

Another driving force to develop XRR methods on curved surfaces is a demand for fast XRR measurements. In this case, a curvature in the flat sample is induced deliberately to obtain a fan of beams reflected at different grazing angles from the sample surface, allowing simultaneous measurements of the scattering intensity with a large vertical size of the beam (20 to 100 µm), in a short-range of *q*_*z*_ using linear (1D) (Stoev & Sakurai, 2011 ▸, 2013 ▸) or two-dimensional (2D) (Liu *et al.*, 2017 ▸; Festersen *et al.*, 2018 ▸) detectors. However, in these methods, the sample curvature limits the *q*_*z*_ range.

Here, we present an X-ray reflectivity measurement method appropriate for highly curved surfaces scanned in θ–2θ geometry, using micrometre-sized X-ray beams and large incident angle ranges. The proposed method is specifically suitable for *in situ* XRR measurements of the curved surfaces of LMCats. Examples of the XRR measurements of bare and graphene-covered surfaces of liquid copper are presented.

## 2. XRR simulation and data analysis principle

**Figure 1:**
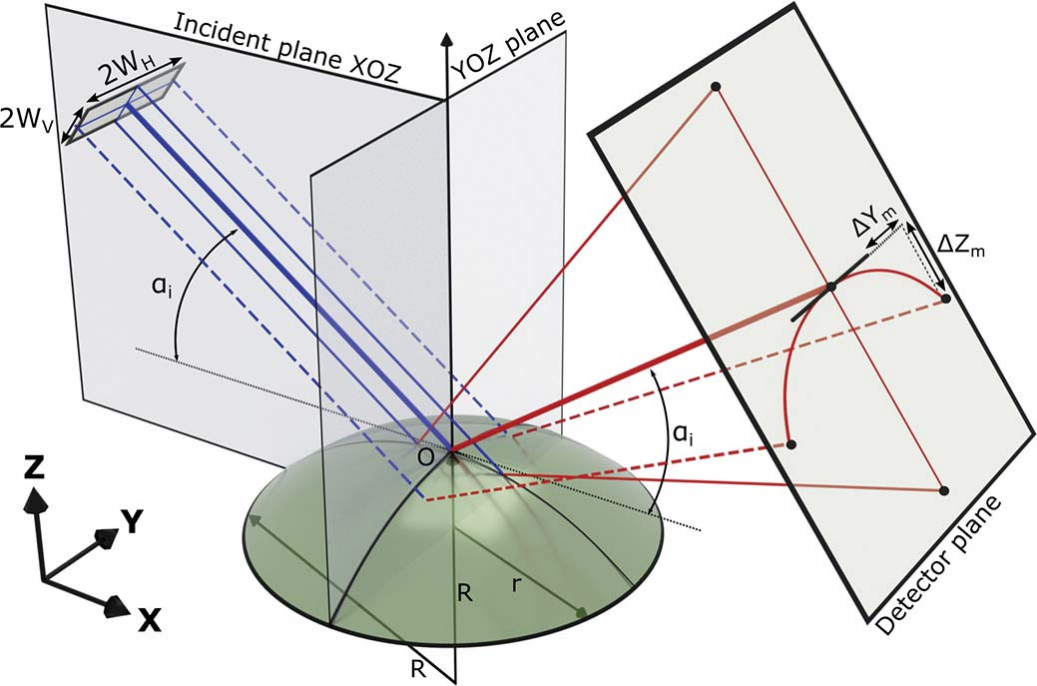
Scheme of the incidence and reflected X-ray beam on a spherical surface with a coordinate system selection. Solid blue and solid red rays represent the primary and reflected rays propagating in the incident plane *XOZ*, while dashed blue and red rays are in the orthogonal plane. A curved surface is considered as a cut from a sphere of radius *R* having base radius *r*. Δ*Y*_m_ and Δ*Z*_m_ indicate the offsets of the ray reflected on the surface curved in the coronal plane in comparison with the reflection on the flat surface.

We consider a sessile drop of liquid placed on a solid substrate with the shape of a spherical dome cut from a sphere of radius *R* and having base radius *r* (Fig. 1 ▸). The surface area illuminated by the X-ray beam, centered on the dome apex, decreases with increasing grazing angle α_i_ between the surface and the beam. In comparison with scanning a curved sample through a nano-focused X-ray beam as illustrated by Festersen *et al.* (2018 ▸), the single-shot measurement method described here provides approximately the scattering from this same area on the sample and in a larger *q*_*z*_ range in comparison with the single-shot measurement using micro-beam mode used by Festersen *et al.* (2018 ▸). The origin of the laboratory coordinate system coincides with the dome apex, point *O* in Fig. 1 ▸. The vertical plane *ZOX* coincides with the main incidence plane. We assume that the incident beam in the cross-section has dimension 2*W*_V_ in the vertical plane and 2*W*_H_ in the horizontal plane (Fig. 1 ▸). The incident beam makes a grazing angle *α*_i_ with the *YOX* horizontal plane (Fig. 1 ▸). When a parallel beam of small size reflects on the spherical surface, it spreads vertically and horizontally. Locally, the incident angle of each elementary ray, an infinitely small portion of the beam, differs from the angle *α*_i_ of the reference ray reflected on the apex. The surface curvature at any point is characterized by two radii lying in orthogonal planes coinciding with the surface normal at this point. For the apex point, these are the *XOZ* and *YOZ* planes, the sagittal and coronal planes, respectively. The effect of curvature on the deflection of the reflected beam in the sagittal and coronal planes is different. This effect will be analyzed for each curvature component separately. The curved surface can be represented as a cylinder of radius *R* oriented by its axis along the *OX* axis for curvature in the coronal plane and along the *OY* axis for curvature in the sagittal plane.

Reflectivity on the surface curved in the coronal plane has been described earlier by Briscoe *et al.* (2012 ▸), where the formulas for the reflected beam offsets on the detector plane, Δ*Y*_m_ and Δ*Z*_m_ (Fig. 1 ▸), in comparison with the reflection on the flat surface were obtained. Using these formulas we calculated that for our experimental conditions (*e.g.* a sample-to-detector distance, a curvature radius, beam size, and the pixel size of the 2D detector, which are given later in Section 3) the calculated maximal vertical and horizontal displacements in the whole range of measurements are negligibly small (*dq_z_* < 10^−4^ nm^−1^). From this perspective, as we perform XRR on the liquid copper drops with *R* > 100 mm satisfying condition *W*_H_/*R* < 10^−3^ following Briscoe *et al.* (2012 ▸), without loss of generality, the effect of the coronal curvature can be neglected and excluded from further considerations. In this case, the spatial spread of the reflected signal on the curved surface can be appropriately described by the sagittal curvature alone: a reflection on the cylinder with the axis perpendicular to the incident plane *XOZ*. Thus, the three-dimensional problem can be reduced to a two-dimensional one.

**Figure 2:**
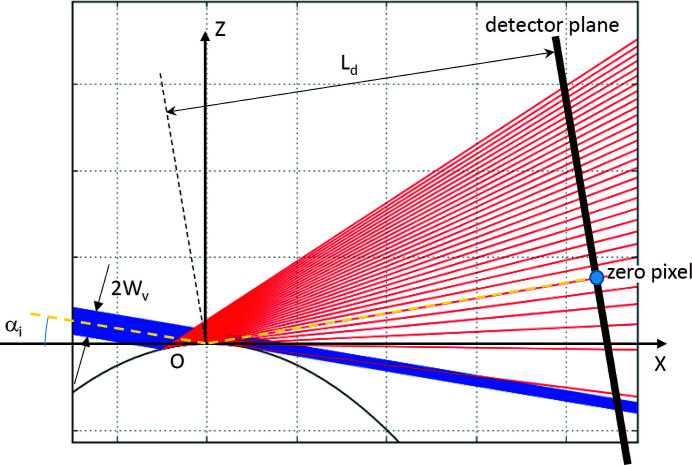
Scattering of the collimated X-ray beam on a cylindrical surface of radius *R*. The incident beam, coming from the left side, propagates in the plane *XOZ* normal to the cylinder axis that is perpendicular to the figure plane. A segment on the left side of the picture represents the detector plane, which is normal to the *XOZ* plane. ‘Zero pixel’ shows the position of the reflected beam from the cylinder apex. ‘Zero pixel’ is the origin of the coordinate system on the detector plane.

**Figure 3:**
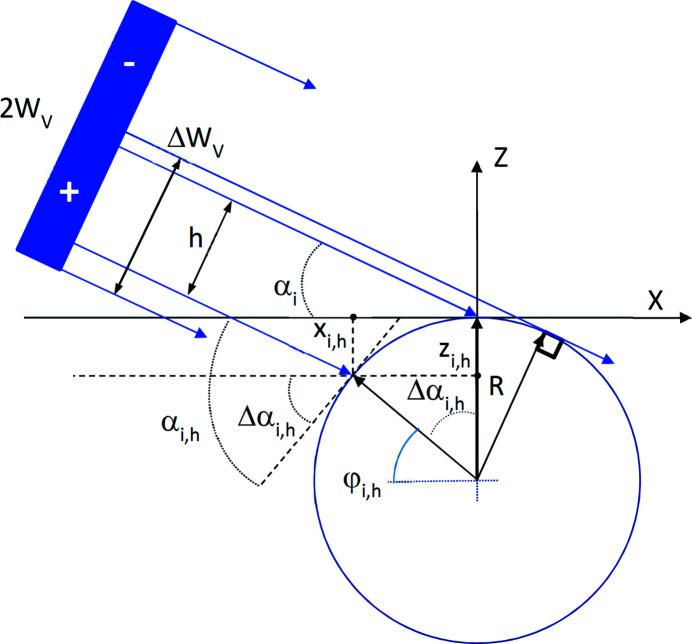
Detailed scheme of the incidence beam with a vertical size 2*W*_V_ as defined by slits before the sample as it impinges on the curved surface with the definition of angles used in formulas. The incident beam comes from the left. Narrow blue lines represent elementary rays. Here, *h* is the offset of the elementary ray from the beam center. This ray impinges with the circular surface of radius *R* at the point with coordinates (*x*_i,*h*_, *z*_i,*h*_). The angle *a*_i_ is the effective grazing angle, while *a*_i,*h*_ and Δ*a*_i,*h*_ are the actual grazing angle at the point (*x*_i,*h*_, *z*_i,*h*_) and the difference of this angle from *a*_i_, respectively.

Let us examine the X-rays reflected by a cylinder where the incident beam propagates in the plane *XOZ* normal to the cylinder axis, and the center of the beam hits the cylinder apex at an effective grazing angle *α*_i_ (Fig. 2 ▸). The effective grazing angle is the angle between the incident beam and the tangent plane (*XOY*) at the cylinder apex. The considerations presented in the previous paragraph allow us to integrate both the incident and reflected beam intensities along the *Y*-direction and to project the integrated intensity of the corresponding effective ray onto the *XOZ* plane. Fig. 2 ▸ shows a beam of vertical size *2W*_V_, hitting a cylinder at an effective angle *α*_i_. At a certain range of *α*_i_, the upper part of the beam, above the central line, does not illuminate the surface and propagates further without being scattered and might hit the bottom of the X-ray detector (Fig. 2 ▸). The rest of the beam is reflected by the curved surface with an angular spread (red lines in Fig. 2 ▸) and is recorded by the detector. Fig. 3 ▸ illustrates the described situation in more detail. The reflected part of the beam Δ*W*_V_ can be calculated using

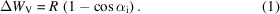

The minimal angle *α*_i,m_, at which the beam is fully reflected from the surface, can be calculated assuming Δ*W*_V_ = *W*_V_ and using equation (1), 

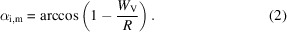

As, by definition, the central ray hits the surface at the angle *α*_i_, the ray shifted from the central ray by a distance *h* will hit the surface with a grazing angle α_i,*h*_ = α_i_ + Δα_i,*h*_ (Fig. 3 ▸), where

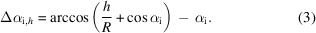

**Figure 4:**
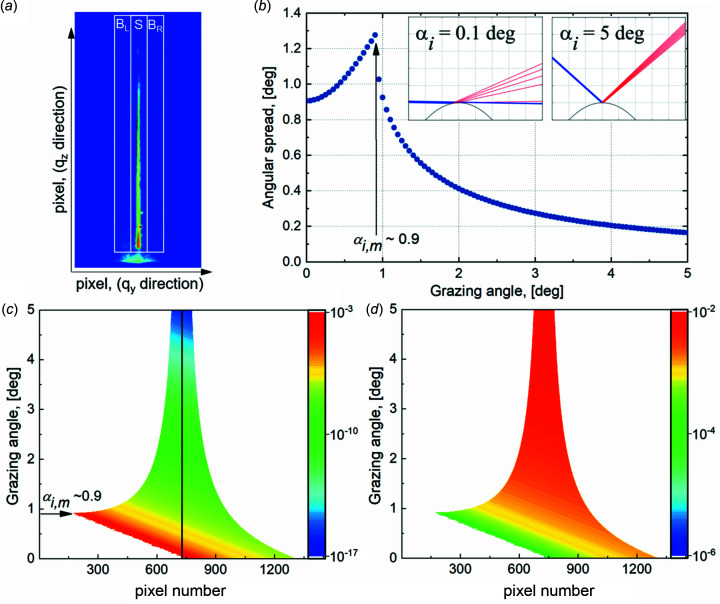
(*a*) Example of a fan of the reflected beam from the curved surface of the molten copper (Sample 3, ID10/ESRF data). Three white rectangles of equal area indicate the areas for calculation of the background (B_L_, B_R_) and the scattered signal (S) for each horizontal cut through the image. A halo below the white rectangles is the parasitic scattering of the primary beam. (*b*) Simulated angular spread value of the beam on a curved surface with a curvature radius of 200 mm and a vertical beam size of 50 µm. Inserts demonstrate the angular spread at effective grazing angles of 0.1 and 5° (from left to right) for the beam represented with nine rays and seen on a detector plane located at 1 m (scale of the vertical grid is 1 mm). In the case of 0.1°, only five rays reflect, and the four others go above the sample, while, in the case of 5°, all nine rays reflect from the sample surface. (*c*) 2D map of simulated X-ray reflectivity on the curved surface for a set of effective grazing angles. The ordinate of the map is the effective grazing angle α_i_, while the abscissa is pixels of the effective 1D detector. The horizontal line at each α_i_ represents the reflected beam intensity distribution along the 1D profile in the *XOZ* plane. Each pixel on the map corresponds to a local grazing angle α_i,*h*_. Parameters of the calculation: radius of the curved surface of liquid copper at *T* = 1400 K is 200 mm; incident beam size (2*W*_V_) is 50 µm; detector pixel size is 55 µm, and sample-to-detector distance is 1.0 m. The vertical line at pixel 728 is a guide to the eye of the effective 1D detector center where α_i,*h*_ = α_i_. The beam’s intensity reaching the detector without reflection is not added to the graph. The color bar corresponds to the logarithm of intensity. (*d*) 2D map of the incident beam contribution fraction to the scattering in a pixel. These values are used for pixel-by-pixel normalization of intensity in Fig. 4 ▸(*c*).

As indicated in Fig. 3 ▸, *h* has a positive value for rays below the central line and negative for rays above the line. The angular spread of the reflected beam is in the range from 0 (for α_i_ < α_i,m_) or α_i_ + Δα_i,–*W*_ (for α_i_ > α_i,m_) to α_i_ + Δα_i,+*W*_ (for *W* < *R*). Equation (3) applied to the experimentally measured angular spread [Fig. 4 ▸(*a*)] can be used to calculate the surface curvature. Fig. 4 ▸(*b*) presents the simulated angular spread of the 50 µm-sized beam reflected by a curved surface with a radius of 200 mm. The highest spread is calculated at α_i,m_. For a sufficiently high *W*_V_/*R* ratio, a complete reflectivity curve can be recorded in one shot, as performed by Festersen *et al.* (2018 ▸) in a short *q*_*z*_ range down to the reflectivity level 10^−5^. An angular range from 0 to 180°, the maximal range, can be reached when *R* = *W*_V_. With increasing grazing angle, the angular spread becomes smaller, approaching asymptotically a value calculated for a flat surface reflection [Fig. 4 ▸(*b*) insets]. It is interesting to look at the opposite extreme case when the beam spread fan is large. With a ratio *W*_V_/*R* = 0.002 (*i.e.* *W*_V_ = 0.025 mm and *R* = 12 mm), the angular spread reaches 5.1°, corresponding to a *q*_*z*_ range of 2 Å^−1^ for an X-ray beam energy of 22 keV. XRR can be obtained in this *q*_*z*_ range in one shot. Let us assume that the reflected beam is recorded with a 1D detector with 100 pixels of 55 µm in the *XOZ* plane. As a rough approximation, we can say that only one-hundredth of the initial beam reflects to a pixel. Novel synchrotrons provide monochromatic X-ray beams with fluxes on the sample of 10^12^ photons s^−1^ or higher; thus, even a hundredth part of this flux allows to measure the reflectivity in one shot, potentially down to 10^−10^, which is still better than reflectivity measurements using a laboratory X-ray source. In the above example, the scattering vector resolution of Δ*q*_*z*_ = 0.02 Å^−1^ allows for suitable studies of thin films with thicknesses below 6 nm. Assuming the total flux of the X-ray beam is 10^12^ photons s^−1^ and only 10 photons s^−1^ are registered in a pixel corresponding to the highest *q*_*z*_ (∼0.7 Å^−1^), hundreds of seconds of counting time can provide the reflectivity curve in one shot in the range *q*_*z*_ ≤ 0.7 Å^−1^ with a statistical error less than 3%.

Equation (3) calculates the reflected intensity recorded by each 1D or 2D detector pixel. With Fig. S3 of the supporting information, the procedure of intensity calculation is the following. The X-ray beam of size 2*W*_V_ and intensity *I*_o_ is divided into *N* equally spaced elemental rays with intensity *I*_o_/*N*. For a given curvature radius and an effective grazing angle α_i_, one can calculate the local grazing angle α_i,*h*_ and the coordinates (*x*_i,*h*_, *z*_i,*h*_) of each elemental ray on the cylindrical surface. Knowing that the specularly reflected and incident beam have this same angle with the surface, only different in sign due to the mirror reflection, one can calculate the vector direction of each reflected elemental ray,

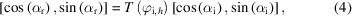

where 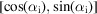 is the incident beam vector. *T*(φ_i,*h*_) is the mirror reflection matrix around the line defined by the circle center and point (*x*_i,*h*_, *z*_i,*h*_) and having the inclination angle φ_i,*h*_ with the *X*-axis (Fig. 3 ▸). The coordinates of this point define the angle φ_i,*h*_ and the matrix *T*(φ_i,*h*_),

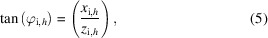




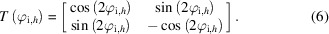

The direction vector of the reflected beam and the (*x*_i,*h*_, *z*_i,*h*_) coordinates are sufficient to calculate the cross-section point on the detector plane. In all presented cases, the detector plane is at a distance *L*_d_ from the apex of the curved surface, and the detector rotates around this point following the effective grazing angle (α_i_:2α_i_) so that the ray reflected from the apex (representing the horizontal plane) arrives at the same point on the detector at any α_i_. This point is considered as the position of the so-called ‘zero pixel’ of the detector (Fig. 2 ▸). The intensity *I*(α_i,*h*_) of the elemental ray after reflection is equal to the theoretical reflectivity *R*(α_i,*h*_) at angle α_i,*h*_ multiplied by the elemental ray intensity *I*_o_/*N* so that

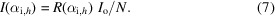

However, equally spaced elemental rays before reflection are unequally spaced on the detector after being scattered on the surface (Fig. 2 ▸), so that the obtained reflected intensity has to be rebinned following the pixel size of the detector array and the portion of the initial beam (*I*_o_) reflected into each pixel. The center of the pixel is used to calculate the corresponding scattering vector,

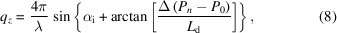

Δ being the pixel size and *P*_0_ and *P*_*n*_ the numbers of the pixels for zero position and reflection position, respectively. A result of such a calculation for a series of α_i_ is presented in Fig. 4 ▸(*c*) in the form of a combined 2D map. Each horizontal line of the plot represents the reflected beam intensity distribution along the 1D profile in the *XOZ* plane for a given incident angle α_i_. This 1D profile we can consider as an effective 1D detector. Calculations are performed for a surface with a curvature radius of 200 mm, a beam size 2*W*_V_ = 50 µm, a pixel size of 55 µm, and a sample-to-detector distance of 1 m. The values of the density and the surface tension of liquid copper at temperature *T* = 1400 K are 7.96 g cm^−3^ and 1.3 N m^−1^ (Harrison *et al.*, 1977 ▸). The calculated surface roughness of the liquid copper caused by the capillary waves can be in the range 1.5–2 Å depending on the used formulas (Braslau *et al.*, 1988 ▸; Mora & Daillant, 2002 ▸; Shpyrko *et al.*, 2003 ▸). For the reflectivity simulation, we used a surface roughness value of 1.5 Å following Shpyrko *et al.* (2003 ▸) (see Section S1 of the supporting information). Fig. 4 ▸(*c*) clearly shows that (at least above a given angle of α_i,m_ ≃ 0.9° here) the angular spread narrows with increasing α_i_, as discussed earlier [see Fig. 4 ▸(*b*)]. This variation of the angular spread with α_i_ can be used to precisely determine the surface curvature (see Section S2 of the supporting information). Iso-color lines on the map relate to the reflected intensity with equal scattering vector *q*_*z*_.

**Figure 5:**
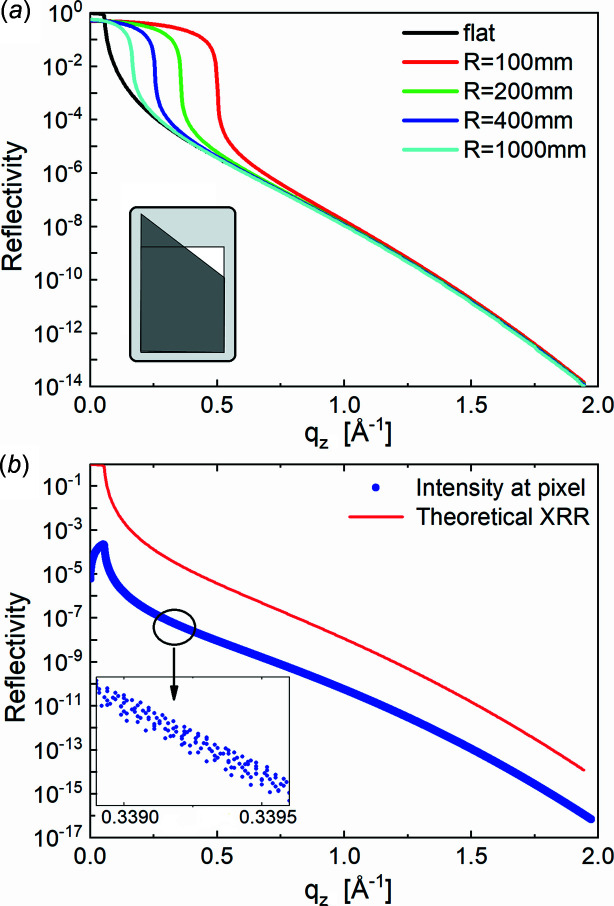
(*a*) Approximated reconstruction of the reflectivity curve (green line) using the 2D map of Fig. 4 ▸(*c*) and the detector integration approach (integration along horizontal lines) for the case of a curvature radius *R* = 200 mm and a beam size 2*W*_V_ = 50 µm. The black curve corresponds to the theoretical XRR on the flat surface (*R* = ∞). The effect of *W*_V_/*R* on the reconstructed reflectivity curve calculated at *W*_V_ = 25 µm for a series of surface curvatures with radius *R* is demonstrated with the colored curves. The interval, where colored curves coincide with the curve calculated for the flat surface (black curve), corresponds to the interval, α_i_ > 2α_i,m_, where the effect of the curvature can be neglected. Inset: geometrical illustration of the equivalence of the integrated reflectivity intensity on the flat and curved surfaces at the effective grazing angle above 2α_i,m_. The rectangle represents the integrated reflected intensity on the flat surface at the effective grazing angle α_i_. The right-angled trapezoid represents the integrated reflected intensity on the curved surface also at α_i_. (*b*) Reflectivity intensity at each pixel (blue dots) of the 2D map in Fig. 4 ▸(*c*). The red curve is a theoretical XRR curve on a flat surface. Insert: zoom of a small part of the graph with measured intensity at *q*_*z*_ around 0.3393 Å^−1^; at this magnification, the blue curve consists of dots.

The calculated 2D map of Fig. 4 ▸(*c*) demonstrates the feasibility of reflectivity curve reconstruction from the presented data. An approximated approach consists of integrating the full intensity along the 1D profile at each effective grazing angle, *i.e.* all reflected beams and the part of the direct beam that reaches the detector without touching the sample (rest of the direct beam). The resulting integrated curve for the curvature radius *R* = 200 mm, together with the theoretical reflectivity curve calculated for the flat surface (*R* = ∞), is shown in Fig. 5 ▸(*a*). Note that the reconstructed and theoretical curves coincide at *q*_*z*_ above 0.6 Å^−1^, corresponding to the effective grazing angle 2*α*_i,m_. The matching of the curves can be explained by the small variation of the grazing angles (and hence *q*_*z*_) under the beam footprint for α_i_ > 2*α*_i,m_ at the corresponding *W*_V_/*R* ratio. As a result, integrating the reflectivity signal with the linear-like variation of the intensity gives the same value as it is scattered from the flat surface at an effective grazing angle *α*_i_. The geometrical interpretation of this effect is illustrated by the insert in Fig. 5 ▸(*a*). The significant mismatch between the reconstructed and expected curves at small *q*_*z*_ values (*q*_*z*_ < 0.6 Å^−1^) is caused by the considerable variation of grazing angles ranging from the total external reflection region to several critical angles, θ_c_, where the reflection intensity drops by several orders of magnitude. Moreover, only a part of the entire incident beam in this region hits the sample. As a result, the integrated intensity in the region of total reflection, *q*_*z*_ < *q*_c_ = 4πλ^−1^sin(θ_c_), becomes lower than the expected reflection, *R*(*q*_*z*_) ≃ 1, from the flat surface. The abrupt drop of intensity at *q*_*z*_ ≃ 0.35 Å^−1^ corresponds to the angle α_i,m_ above which the entire incident beam hits the sample surface. According to equation (2), the α_i,m_ value depends on the *W*_V_/*R* ratio. For *R* = 200 mm, 2*W*_V_ = 50 µm and X-ray energy 22 keV, used for Fig. 4 ▸(*c*) and Fig. 5 ▸(*a*) calculation, one can obtain α_i,m_ ≃ 0.9° and correspondingly *q*_*z*_ ≃ 0.35 Å^−1^. Reconstructed reflectivity curves for several *W*_V_/*R* ratios are shown in Fig. 5 ▸(*a*), clearly showing that the ideal XRR curve of a flat surface is slowly approached with increasing *R* or, more precisely, decreasing *W*_V_/*R* ratio.

Hereafter we present an accurate description of the XRR curve reconstruction based on the intensity analysis pixel by pixel with a proper normalization to the incident intensity. Fig. 5 ▸(*b*) shows the reconstructed reflectivity of a flat surface with the intensity distribution for each *q*_*z*_ (corresponding to the pixel center) along with the effective 1D detector, as shown in Fig. 4 ▸(*c*). The inset in Fig. 5 ▸(*b*), the zoom-in of the smaller distribution range, aims to show that there are multiple points in the vicinity of any selected *q*_*z*_ value. The calculated blue curve is below the theoretical one because the reflected intensity at a pixel is not yet normalized on the incident beam contributing to the pixel. Equation (7) shows that only a small part of the initial beam is reflected towards each pixel. Although the pixel is illuminated by a small part of the entire beam, the averaged intensity at a given *q*_*z*_ improves the measurement statistics, *i.e.* the error bar of the intensity reconstructed for a given *q*_*z*_ decreases. The ideal, normalized XRR curve is simply recovered by dividing the intensity at each pixel by the corresponding fraction of the entire incident beam [Fig. 4 ▸(*d*)], the inverse of equation (7) *R*(α_i,*h*_) = *I*(α_i,*h*_)*N*/*I*_o_. This curve perfectly coincides with the reference curve from the flat surface and is not shown in Fig. 5 ▸(*b*).

## 3. XRR measurements and materials

After reconstruction of the XRR using simulated data, the exact method was applied to two types of real samples: (1) two cylindrically curved solid samples of known curvature *R* = 10.3 mm (Sample 1) and *R* = 516 mm (Sample 2), and (2) a naturally curved liquid surface of copper without (Sample 3) and with (Sample 4) a layer of graphene grown on top.

Samples 1 and 2 are made of glass and have the shape of a horizontal cylindrical segment of length 30 mm. The highly polished surface of the cylinder was coated uniformly with a thin film of gold using magnetron sputtering deposition. The expected thickness of the deposited layer is about 20 nm. XRR of these samples was measured at the ID10 beamline of the ESRF.

The bare liquid copper sample (Sample 3) and the liquid copper with an as-grown graphene layer on top (Sample 4) were prepared and measured *in situ* in a specially designed CVD reactor for 2D material growth on liquid metal catalysts (Saedi *et al.*, 2020 ▸). Three pieces of Cu foil with 12 mm diameter and a thickness of 50 µm were melted on a thick tungsten disk holder. Copper foils with purity 99.9976% from Advent Research were used. The mirror-like polished surface of the tungsten disk, mounted on the heating plate of the reactor, was chemically treated in the central part (diameter 15 mm) to obtain a surface roughness optimal for minimization of the effective contact angle of liquid copper on the rough tungsten following Wenzel’s equation (Wenzel, 1936 ▸). On the polished part, the large W–Cu wetting angle of ∼25° prevents the molten copper from leaking out of the support disk, and the right amount of copper on the rough part provides a small wetting angle (*i.e.* a big radius of curvature). The curvature radii of different samples were varied from 100 mm to 800 mm. An example of the surface curvature after cooling the sample to room temperature (thus solidifying the Cu) is given in Fig. S1. XRR on molten copper was measured at two synchrotron facilities: at the P08 beamline of PETRA III using the LISA instrument (Murphy *et al.*, 2014 ▸; Seeck *et al.*, 2012 ▸) and at the ID10 beamline of the ESRF (Smilgies *et al.*, 2005 ▸). Both instruments are equipped with a double-crystal deflector (DCD), required for studies on liquid surfaces. The DCD steers the X-ray beam down from the horizontal plane, enabling to change the incident beam angle on the liquid surface without moving the sample.

The XRR measurements using LISA were performed with the following experimental settings. The collimated, monochromatic 18 keV X-ray beam at the sample position was 200 µm horizontally and 40 µm vertically in size. An X-Spectrum Lambda-750k detector with a GaAs sensor and 55 µm × 55 µm pixel size was used to measure the reflected beam from the liquid surface. The detector position followed the incident beam so that the reflected beam at the effective angle α_i_ was always reflected to this same pixel. The sample-to-detector distance was 1085 mm.

At the ID10 surface scattering endstation, reflectivity measurements were performed using 22 keV light, monochromated with a Si(111) channel-cut monochromator. The X-ray beam was focused by 32 parabolic beryllium refractive lenses with a curvature radius of 200 µm, and its size at the sample position was 30 µm horizontally and 20 µm vertically. The X-ray beam reflected on the surface was recorded using a 2D photon-counting area detector [Maxipix (Ponchut *et al.*, 2011 ▸), of 55 µm × 55 µm pixel size, 28.4 mm × 28.4 mm detection area, and using a CdTe sensor of 1 mm thickness] placed at a distance of 488 mm. A total counting time of 8 min for recording the scattering signal was sufficient to reconstruct the XRR curve in the *q*_*z*_ range from 0 to 2 Å^−1^.

The XRR measurements on liquid copper were performed above the copper melting temperature (1357.77 K) at *T* = 1400 ± 10 K, with a gas mixture (Ar 91%, H_2_ 9%) at 0.2 bar pressure in the reactor. A graphene layer on liquid copper was grown under the conditions described by Jankowski *et al.* (2021 ▸), namely *T* = 1400 K, 0.2 bar, gas mixture Ar 91.24%, H_2_ 8.69%, and CH_4_ 0.06%, and with a 230 standard cubic centimetres per minute (sccm) flow rate. The XRR measurements were started when graphene covered the entire surface of the liquid copper. The growth coverage was monitored in real time with radiation-mode optical microscopy (Terasawa & Saiki, 2015 ▸; Jankowski *et al.*, 2021 ▸).

## 4. Results and discussion

**Figure 6:**
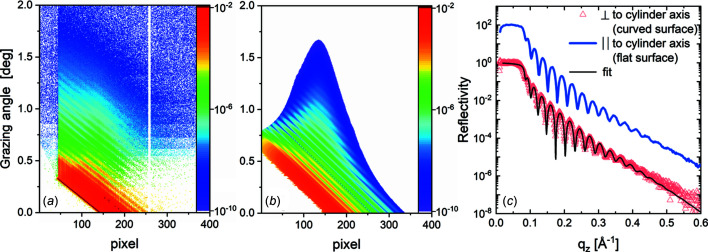
XRR results for solid cylindrical surfaces. Measured (*a*) and simulated (*b*) 2D scattering pattern of thin gold film on the cylinder with radius *R* = 516 mm (Sample 2). (*c*) XRR curves measured along the cylinder axis (blue line), reconstructed from reflection on the cylinder axis oriented perpendicular to the incident beam (red dots) and corresponding calculation with the slab model (black line). The blue curve is shifted by two orders of magnitude for clarity.

**Figure 7:**
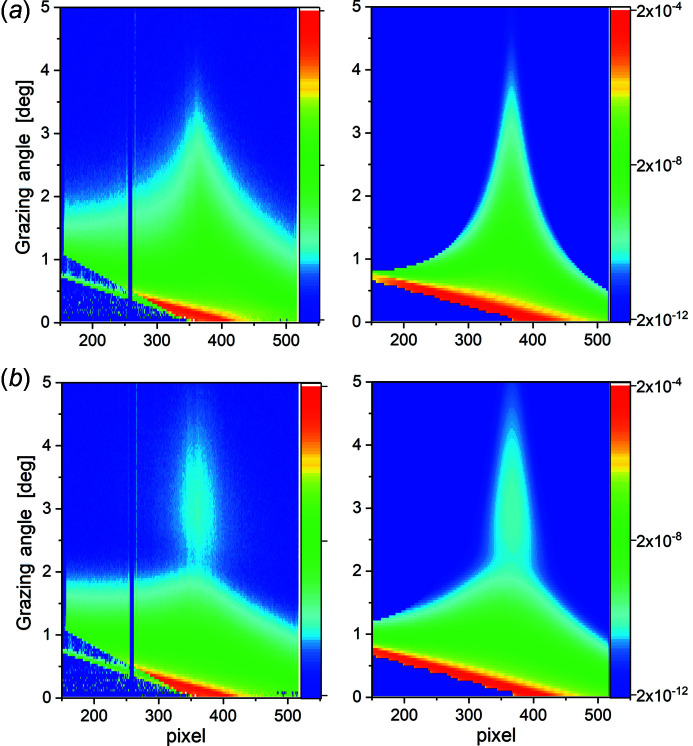
(*a*) 2D map of liquid copper measurement (left) and the corresponding calculation (right) with the curvature radius *R* = 215 mm; (*b*) 2D map of liquid copper with graphene: measurement (left) and corresponding calculation (right) with the curvature radius *R* = 115 mm.

**Figure 8:**
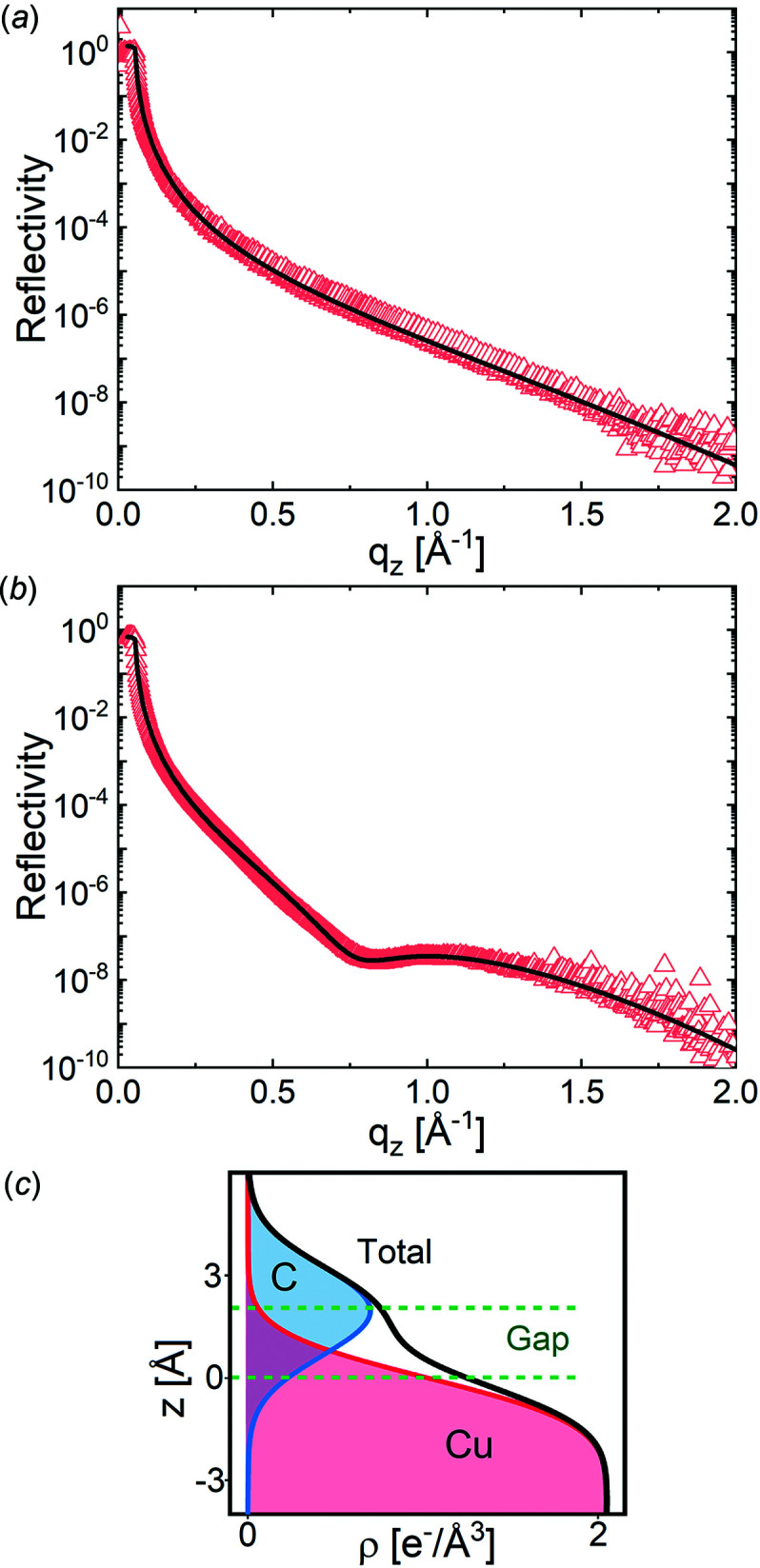
Reconstructed XRR curves (triangles): (*a*) liquid copper and (*b*) liquid copper with graphene. The solid line is the best fit. (*c*) Resulting electron density model of the graphene layer on the liquid copper split into the contributions of different components and the separation gap. The separation gap is defined as Gap = *t*_S_ + *t*_G_/2.

The scattering intensity measured with the 2D detector at each effective grazing angle was transformed to a 1D profile lying in the plane *XOZ* by integration and background subtraction of the signal along the direction perpendicular to *q*_*z*_. The reflected intensity *R* ^*i*^ at each point *i* of the new 1D profile is obtained from *R* ^*i*^ = 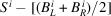 , where *S* ^*i*^, 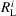 , 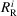 are the signal and background contribution on both sides of the cut-through *q*_*z*_ direction [Fig. 4 ▸(*a*)]. The resulting 2D maps normalized to the entire intensity of the incident beam are presented in Figs. 6 ▸(*a*), 7 ▸(*a*), and 7 ▸(*b*). Both maps (Fig. 7 ▸, left column) have a systematic decrease of the angular spread of the X-ray beam with increasing grazing angle values, following the prediction [Fig. 4 ▸(*c*)]. In comparison with the reflection on the bare copper [Fig. 7 ▸(*a*)], the reflection on the copper with graphene layer demonstrates an intensity oscillation [Fig. 7 ▸(*b*)], an increase of intensity after a small decrease, caused by the interference of the beam scattered from the film interfaces separated by a thickness *t*. The nonideal coincidence between measured (left column in Fig. 7 ▸) and calculated (right column in Fig. 7 ▸) maps is caused by the background scattering aside of the specular beam and the non-Gaussian beam shape far from its center. A Gaussian beam shape was used for the maps simulation. To avoid the problems from these effects, we used only the central part (FWHM) of the reflected beam for further analysis. Within the FWHM of the reflected beam, the scattering intensity matches with the calculated intensity, as demonstrated in Fig. S2. The intensity at each pixel of these maps, normalized to the intensity of the incident ray contributing to this pixel, following the method described previously and plotted versus corresponding *q*_*z*_ value [Figs. 8 ▸(*a*) and 8(*b*) and Fig. 6 ▸(*c*) dots], gives the required XRR curves, as they would be measured on the flat surface. There are many points on this graph with very similar *q*_*z*_ values. If necessary, these points can be averaged or re-binned on a new grid to reduce the statistical error of the integrated XRR signal. The obtained reflectivity curves are used for further analysis to extract structural information of the samples. It is worthwhile noting here that the XRR measurement performed on curved surfaces with a ratio *W*_V_/*R* < 10^−4^ allows for the reconstruction of the reflectivity curve using the approximate procedure, integration of the scattered signal over the entire detector, and attribution of this intensity to the effective *q*_*z*_ value [Fig. 5 ▸(*a*)]. XRR curves, obtained in this way, are similar to the XRR curves measured on the flat surface for *q*_*z*_ > 0.5 Å^−1^ (in the case of *R* ≥ 200 mm).

Fig. 6 ▸ presents the XRR results on the solid cylindrical surface for Sample 2. For simplicity of the narrative, the results for Sample 1 are shown in Fig. S3. Fig. 6 ▸(*a*) shows the experimental data, Fig. 6 ▸(*b*) shows the simulated data, and Fig. 6 ▸(*c*) shows the reconstructed XRR data. Diagonally inclined periodic oscillation of the intensity in Figs. 6 ▸(*a*) and 6(*b*) corresponds to the Kiessig fringes oscillations caused by the interference of the X-ray waves reflected on the interfaces of the thin gold film, so-called thickness oscillation. The more extensive angular spread of the reflected beam on the cylinder with the smaller curve radius is illustrated by measurements on Sample 1 and Sample 2 (Fig. 6 ▸ and Fig. S3). The absence of the abrupt intensity drop on the measured 2D maps [Fig. 6 ▸(*a*)] compared with the calculations [Fig. 6 ▸(*b*)] is explained by the absence of the statistical noise and the background on the calculated data. A beam profile with Gaussian shape was used for the calculations. The beam profile parameters correspond to the measurement of the primary X-ray beam profile. Reflectivity measurements were performed with two orientations of the cylinder axis: one with the axis perpendicular to the incident X-ray beam and another with the cylinder axis along the beam. The latter measurement is used as a reference for the scattering on the flat sample. The curvature effect is negligible within the 30 µm horizontal beam size as described before. Reconstructed XRR curves for both curvatures are shown in Fig. 6 ▸(*c*) with red triangles. Apart from the small-angle region below *q*_*z*_ = 0.05 Å^−1^, both curves, measured along and perpendicular to the cylinder axis, match [blue and red curves in Fig. 6 ▸(*c*)]. The reflectivity is higher than 1 for *q*_*z*_ < 0.05 Å^−1^ (grazing angle < 2 mrad), explained by the difficulties with the reflected intensity normalization to the intensity of the incident elemental ray, whose size becomes extremely small. The black curves in Fig. 6 ▸(*c*) show the best fit of reconstructed XRR curves. XRR curves were fitted using the *REFL1D* program (Doucet *et al.*, 2018 ▸). A simple slab model with one layer was used. Parameters of the model were the density of the glass substrate (ρ_g_), gold film density (ρ_f_) and thickness (*t*_f_), and the roughness of the substrate–film (σ_g_) and film–air interfaces (σ_f_). Model parameters of the best fit are presented in Table 1 ▸.

After the successful test of the exact method of the XRR reconstruction on the curved surface with known curvature, we applied it to *in situ* studies on the curved liquid metal at 1400 K. The XRR curves of the bare liquid copper surface and liquid copper with CVD-grown graphene covering the entire surface were measured initially at the LISA/P08/PETRA-III endstation (Jankowski *et al.*, 2021 ▸) and later, on a more extensive *q*_*z*_ range, at the ID10/ESRF endstation. Therefore, only measurements from ID10 are shown in this publication as the results obtained on the two synchrotron stations are similar. The obtained XRR curves were fitted as before using the *REFL1D* program.

The XRR of the bare liquid copper surface [Fig. 8 ▸(*a*) triangles] can be modeled with two parameters only: the electron density (ρ_Cu_) of the liquid copper at *T* = 1400 K and the roughness (σ_Cu_) at the copper–gas interface. Actually, we fitted only the roughness value, while the electron density of liquid copper at this temperature, 7.99 g cm^−3^ (Cahill & Kirshenbaum, 1962 ▸), was calculated and kept fixed during the fits. The best fit is shown in Fig. 8 ▸ as the solid line. The obtained roughness value, 1.25 ± 0.1 Å, is smaller than expected from the capillary wave theory (Shpyrko *et al.*, 2003 ▸), 1.5 Å. This difference might be explained by the experimental resolution (Braslau *et al.*, 1988 ▸), which is not taken into account. Another reason for this difference can be the effect of the liquid viscosity on the roughness (Jeng *et al.*, 1998 ▸). Viscosity modifies the upper limit of the scattering vector (*q*_max_) used to integrate the capillary waves spectrum. Following Jeng *et al.* (1998 ▸), *q*_max_ for liquid copper at 1400 K is 1.612 × 10^−2^ Å^−1^ , and the resulting roughness is 1.16 Å. This value is close to the one found.

The XRR curve of the graphene layer on the liquid copper [Fig. 8 ▸(*b*) triangles] has a minimum intensity around *q*_*z*_ = 0.8 Å^−1^. The presence and position of this minimum correspond to the presence of the graphene layer on the copper surface. The experimental XRR curve was fitted with two different slab models (Fig. S4): Model-1 – a layer on the liquid copper [three fitting parameters: layer thickness (*t*_G_), interfacial roughnesses of copper–graphene (σ_Cu_) and graphene–gas interfaces (σ_G_); two fixed parameters: liquid copper (ρ_Cu_) and graphene (ρ_G_) densities]; Model-2 – one carbon atom thick layer separated by a gap from the liquid copper surface [three fitting parameters: the gap thickness (*t*_S_), the interfacial roughnesses of the copper–graphene and the graphene–gas interfaces; three fixed parameters: the liquid copper density, the carbon layer thickness, and the graphene density]. For graphene density, we used the density of a single layer of graphite without a gap between layers.

Model-1, which does not have the separation gap, could not describe the experimental data (Fig. S5, Table S1) within physical constraints on the thickness and density of the graphene layer. Therefore, Model-2 was used. The separation gap (*t*_S_) in this model was defined as the distance between the inflection point of the electron density of the liquid copper and the inflection point of the graphene layer density on the side facing towards copper.

The best fit using the model with the separation gap, Model-2, is presented in Fig. 8 ▸(*b*) and the parameters of the obtained model are given in Table 2 ▸. The graphical presentation of the electron density profile based on these parameters is shown in Fig. 8 ▸(*c*). As in the case of the bare liquid surface, the roughness value is smaller than that expected from the capillary wave theory for the reason discussed above. The slight difference in the roughness of the copper and the graphene layer can be explained by the weak interaction between the graphene layer and the copper (Han *et al.*, 2019 ▸) and by the non-zero bending rigidity of graphene (Lindahl *et al.*, 2012 ▸), which result in the feeble following of the graphene layer to the bending of the copper surface induced by the capillary waves.

## 5. Conclusions

A method for X-ray reflectivity measurements on highly curved surfaces using linear or 2D detectors and a scan of the grazing angle is presented in this work. The mathematical basis of the data analysis is described. This method is applied to a solid surface with known curvature and to the *in situ* study of a CVD-grown graphene layer on molten copper at 1400 K having natural curvature due to the partial wetting of the tungsten substrate. Structural characteristics of the copper surface, the graphene layer, and the separation gap between them are obtained. This method can be applied to any concave curved surface. The scan of the grazing angle increases access to the high values of the scattering angle and, as a result, to the structure resolution.

## Supplementary Material

Supporting Sections S1 and S2; Figures S1 to S5; Table S1. DOI: 10.1107/S1600577522002053/ju5041sup1.pdf

## Figures and Tables

**Table 1:** Results of the fit of the XRR on Sample 1 and Sample 2 Parameters in bold were fixed during the fit.

*R* (mm)	ρ_g_ (g cm^−3^)	σ_g_ (Å)	ρ_f_ (g cm^−3^)	*t*_f_ (Å)	σ_f_ (Å)
10.3	**2.5**	4.4	**19.0**	219.6	7.2
516	**2.5**	4.4	**19.0**	202.9	7.2

**Table 2:** Results of the fit of the XRR on liquid copper with the graphene layer Parameters in bold were fixed during the fit. *t*_G_ is the graphene layer thickness, σ_Cu_ is the roughness of copper–graphene interface, σ_G_ is the roughness of the graphene–gas interface; ρ_Cu_ is the liquid copper density, ρ_G_ is the graphene density, *t*_S_ is the thickness of the graphene–copper separation gap.

ρ_Cu_ (g cm^−3^)	σ_Cu_ (Å)	ρ_G_ (g cm^−3^)	*t*_G_ (Å)	σ_G_ (Å)	*t*_S_ (Å)
**7.99**	1.29 ± 0.09	**5.36**	**1.42**	1.26 ± 0.09	1.55 ± 0.08
