# Supplementary material for: X-ray reflectivity from curved surfaces as illustrated by a graphene layer on molten copper
Source: J Synchrotron Radiat. 2022 Apr 1;29(Pt 3):711–20. doi: 10.1107/S1600577522002053 (PMC9070704; doi:10.1107/S1600577522002053)
Supplement: Supplementary file 1 [file s-29-00711-sup1.pdf]

JOURNAL OF SYNCHROTRON RADIATION

Volume 29 (2022)

Supporting information for article:

X-Ray reflectivity from curved surfaces. Application to a graphene layer on molten copper

Oleg V. Konovalov, Valentina Belova, Francesco La Porta, Mehdi Saedi, Irene M.N. Groot, Gilles Renaud, Irina Snigireva, Anatoly Snigirev, Maria Voevodina, Chen Shen, Andrea Sartori, Bridget Murphy and Maciej Jankowski

S1. Surface roughness calculation

The capillary waves induced surface roughness σ of the liquid copper was calculated using the formula [Shpyrko et al., 2003]

$$\sigma^2 = \frac{k_B T}{2\pi\gamma} log\left(\frac{q_{max}}{q_{res}}\right)$$

In this formula: k_B is the Boltzmann constant, $\gamma = 1.313\ N/m$ is the surface tension of the liquid copper at the temperature $T = 1358\ K$, $q_{max} = 1.9 \cdot 10^{10} m^{-1}$ the value of the upper cutoff for capillary wave contributions defined by the nearest-neighbor atomic distance in bulk, and $q_{res} = 4.1 \cdot 10^5 m^{-1}$instrumental resolution of the measurement. All these values together give the roughness value $\sigma \sim 1.5$ Å.

S2. Curvature determination using experimental or simulated data

Formula 3, where h is replaced by half of the beam size W_V, describes the angular spread of the reflected beam depending on the effective angle α_i and the curvature radius. From the experiment, we know W_V and $\Delta\alpha_{i,W_V}$ for each α_i. Formula 3 resolved for the curvature radius R gives the analytical method of the unknown curvature radius calculation

$$R = \frac{W_V}{cos\left(\Delta\alpha_{i,W_V} + \alpha_i\right) - cos(\alpha_i)}$$

This formula is valid for half of the beam size around the central point. Note that the two parts of the beam have different angular spread after reflection. This difference becomes smaller and smaller with the increase of α_i, as seen in Fig. 4(C). For calculation of the curvature radius, only data with $\alpha_i > \alpha_{i,m}$ must be used ($\alpha_{i,m}$ is defined by formula 2). Better results of the curvature radius determination can be obtained through the fit of the angular spread versus the effective angles α_i. A curve similar to the one shown in Fig. 4(B) is fitted in this case with only one parameter – the radius of the curvature. Figure 4(B) shows the full spread of the beam, $\Delta\alpha_i = \left|\Delta\alpha_{i,+W_V}\right| + \left|\Delta\alpha_{i,-W_V}\right|$, which is measured from the experimental data. It is not trivial to obtain an analytical formula for R in this case, and we define the curvature radius only by the fit of the angular spread for available α_i. The angular spread depends only on the curvature radius and it does not depend on the sample, substrate with a film or without. So there is no difference in the curvature radius determination in these cases.

Table S1 Model parameters of the best fit for the described models with different fixed parameter combinations. Bleu boxes correspond to the parameters fixed during the fit.

model	ρ_{Cu}, g/cm^3	σ_{Cu}, Å	ρ_G, g/cm^3	t_G, Å	σ_G, Å	t_S, Å	Fig.
Model-1 (2-interface)	7.99	σ_G	5.36	1.42	2.39	N.A.	S5C
	7.99	σ_G	5.36	4.26	1.26	N.A.	S5A
	7.99	σ_G	3.52	1.42	1.26	N.A.	S5B
	7.99	σ_G	2.73	4.27	1.26	N.A.	S5D
Model-2 (3-interface)	7.99	1.29 ± 0.09	5.36	1.42	1.26 ± 0.09	1.55 ± 0.08	8B

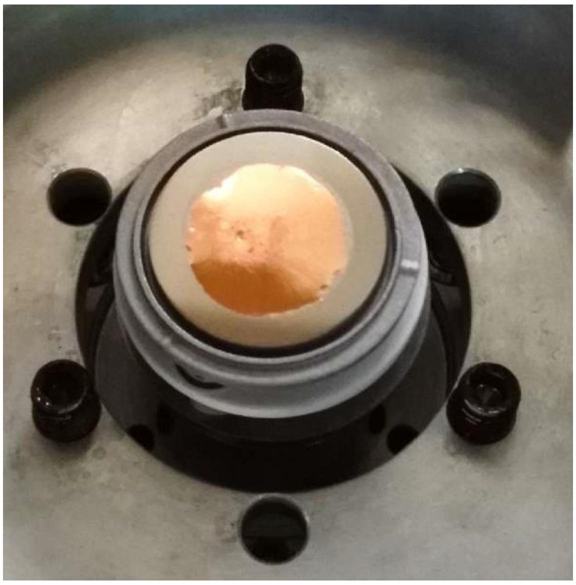

Figure S1 Photo of the copper surface on the tungsten disk and heater after the melting and solidification of a 40 μm-thick copper foil. The diameter of the copper dome is about 10 mm.

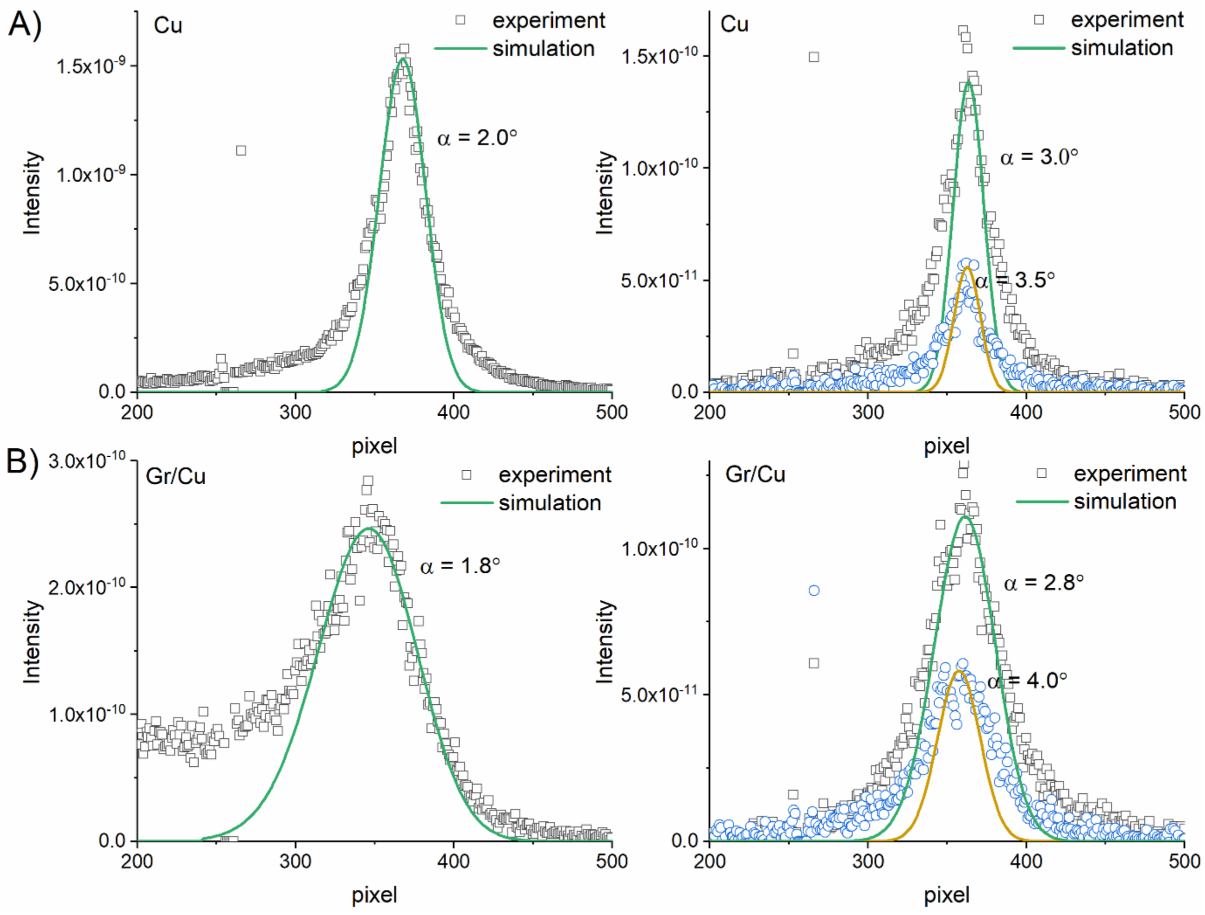

Figure S2 Example of the matching between experimental and calculated 2D maps (see Fig. 7) for bare liquid copper (A) and the liquid copper with the graphene layer (B). Dots (experimental data) and lines (calculated data) represent the horizontal cuts through the 2D maps and some selected effective grazing angles.

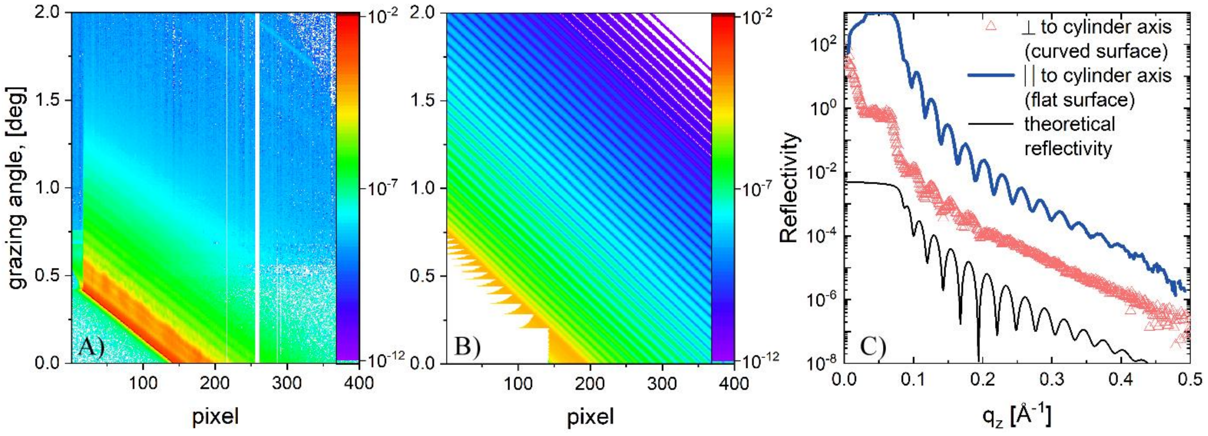

Figure S3 XRR results on solid cylindrical surfaces. Simulated (A) and measured (B) 2D scattering pattern of thin gold film on the cylinder with radius R = 10.3 mm (Sample 1). (C) XRR curves measured along the cylinder axis (blue line), reconstructed from reflection on the cylinder axis oriented perpendicular to the incident beam (red dots) and corresponding calculation with the slab model (black line). For clarity, the blue curve and its fit are offset by two orders of magnitude.

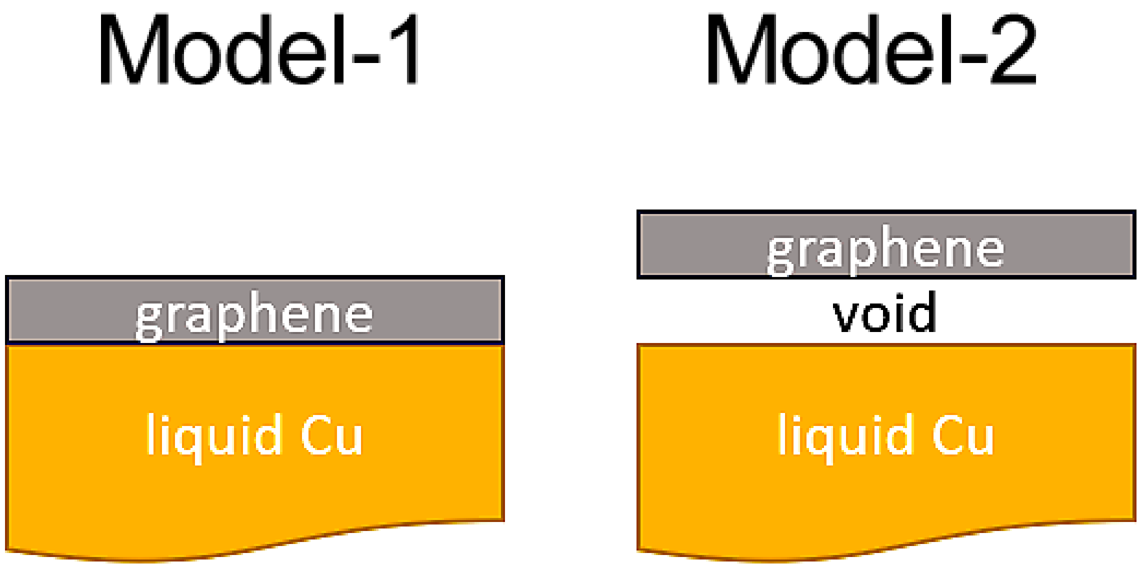

Figure S4 The two models used to fit the Liquid-Cu/Gr experimental data (Sample 4) using the slab models with two interfaces: Model-1 (graphene layer on the liquid copper without a separation gap) and three interfaces, and Model-2 (graphene layer above the liquid copper with a separation gap).

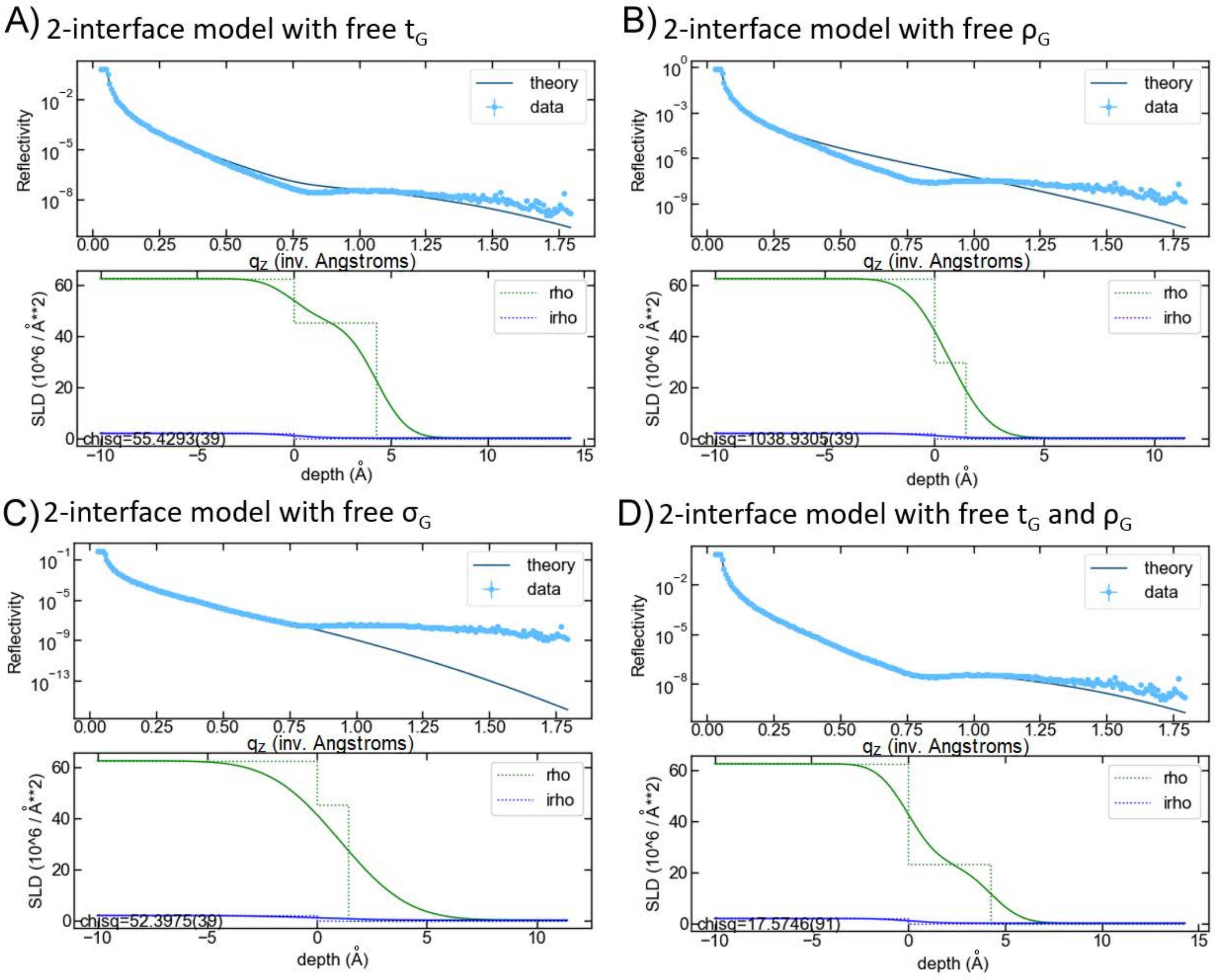

Figure S5 XRR of the experimental data with the best fit and the resulting scattering length density (SLD) following to the Model-1 (two interfaces: liquid-Cu/graphene and graphene/gas) described above and free parameters (Table S1): A) thickness t_G; B) density ρ_G; C) roughness σ_G; and D) two parameters simultaneously t_G and ρ_G. Blue dots are experimental data. Dark blue lines are calculation following to the best model. Real part of the scattering length density (SLD) is represented with the step function (box model) without the interfacial roughness (solid green line) and with the roughness (green dotted line). Curves are shown only for $q_z < 1.75$ Å as already on this interval the difference between the experiment and the calculation is clear.
